# Supplementary material for: Ubiquitous organic molecule-based free-standing nanowires with ultra-high aspect ratios
Source: Nat Commun. 2021 Jun 29;12:4025. doi: 10.1038/s41467-021-24335-x (PMC8241875; doi:10.1038/s41467-021-24335-x)
Supplement: Supplementary file 1 — Supplementary Information [file 41467_2021_24335_MOESM1_ESM.pdf]

Supplementary Information

for

Ubiquitous Organic Molecule-based Free-standing Nanowires with Ultra-High Aspect Ratios

Koshi Kamiya[1], Kazuto Kayama[1], Masaki Nobuoka[1], Shugo Sakaguchi[1], Tsuneaki Sakurai[1]*, Minori Kawata[1], Yusuke Tsutsui[1], Masayuki Suda[1], Akira Idesaki[2], Hiroshi Koshikawa[2], Masaki Sugimoto[2], G.B.V.S. Lakshmi[3], D.K. Avasthi[4], Shu Seki[1]*

[1] Department of Molecular Engineering, Graduate School of Engineering, Kyoto University, Nishikyo-ku, Kyoto 615-8510, Japan.

[2] Takasaki Advanced Radiation Research Institute, National Institutes for Quantum and Radiological Science and Technology, 1233 Watanuki-machi, Takasaki, Gunma 370-1292, Japan.

[3] Special Center for Nanoscience, Jawaharlal Nehru University, New Mehrauli Road, New Delhi 110067, India.

[4] Department of Physics, School of Engineering, University of Petroleum and Energy Studies, Dehradun 248007, India

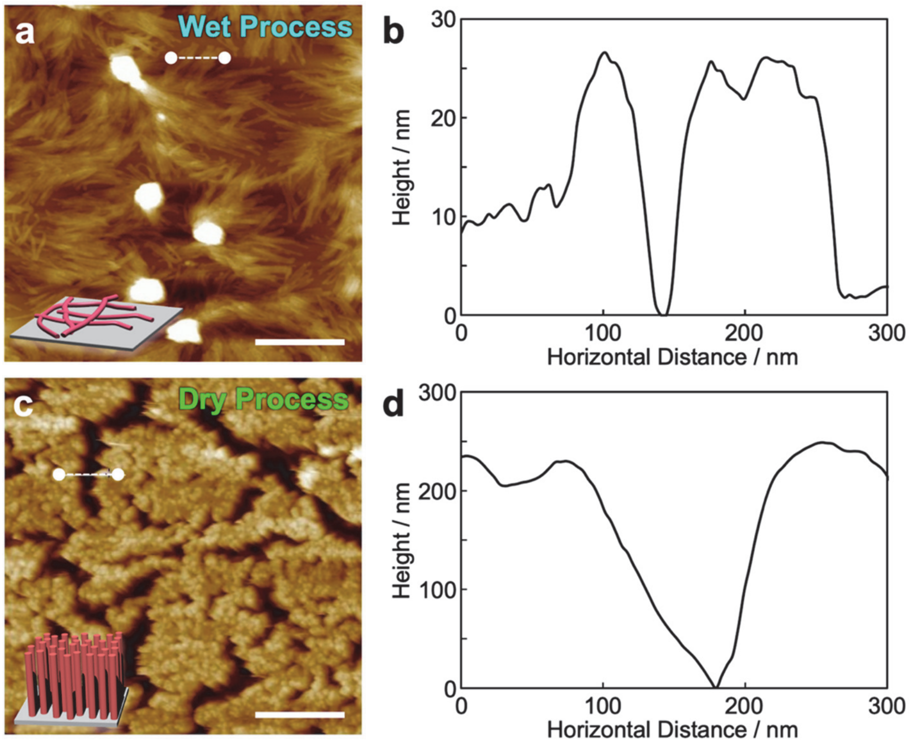

Supplementary fig. 1 | Comparison of morphology of knocked-down and standing nanowires. (a,c) AFM topographic images of nanowires isolated by (a) wet process and (c) dry process. Nanowires were fabricated via irradiation of vapor-deposited 250 nm thickness of C_{60} films with 490 MeV $^{192}Os^{30+}$ at 1.0×10^{11} cm^{-2}. (b,d) Height profiles along white dashed lines in (a) and (c), respectively. Scale bars in (a,c) represent 500 nm.

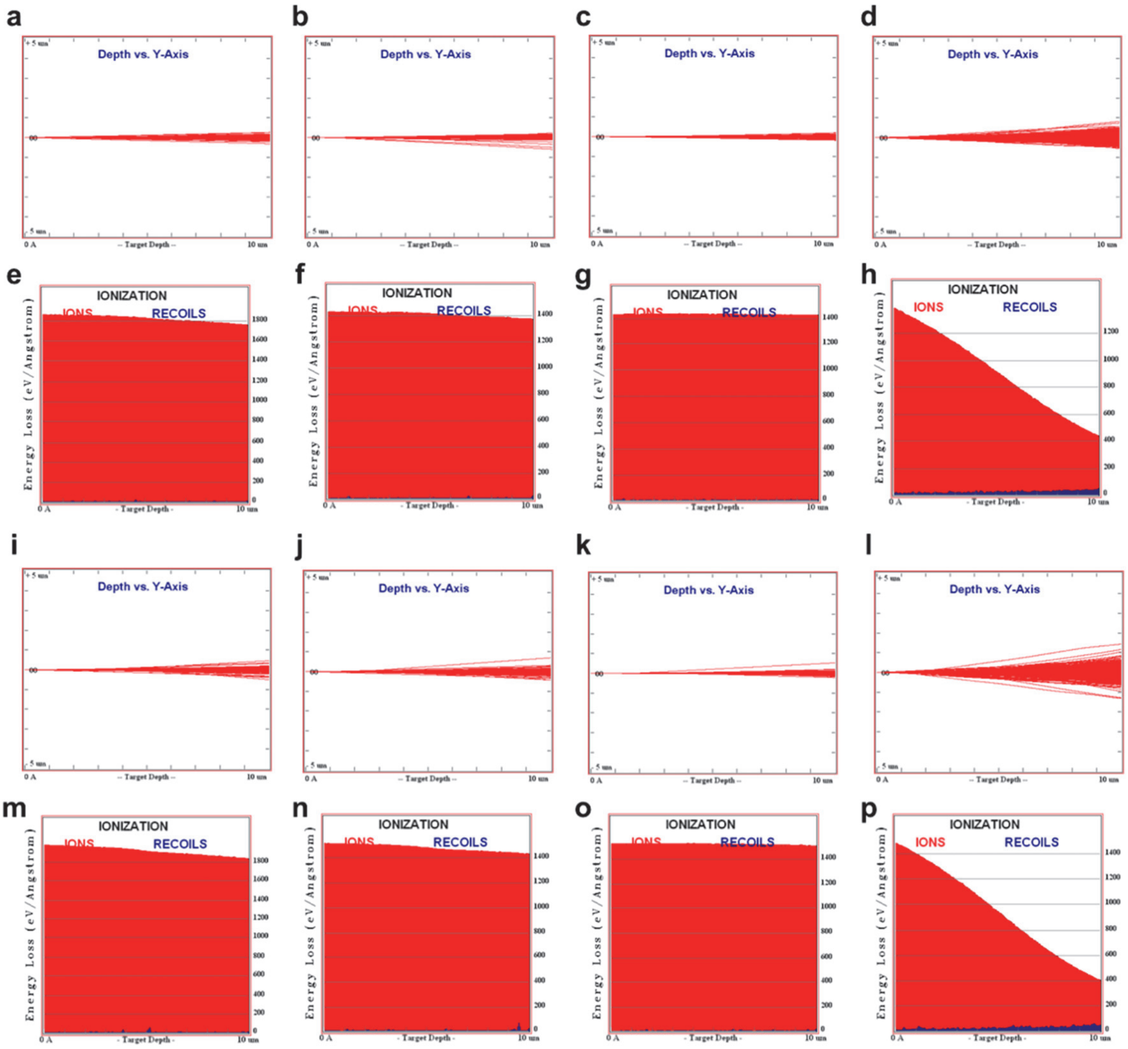

Supplementary fig. 2 | Monte Carlo simulations of ion trajectories. Ion trajectories of (a,i) 490 MeV $^{192}Os^{30+}$, (b,j) 350 MeV $^{129}Xe^{26+}$, (c,k) 450 MeV $^{129}Xe^{23+}$, and (d,l) 120 MeV $^{197}Au^{9+}$ for 10 μm film thickness of (a–d) C_{60} and (i–l) PTCDA-Cl_4. LET vs. target depth profiles of (e,m) 490 MeV $^{192}Os^{30+}$, (f,n) 350 MeV $^{129}Xe^{26+}$, (g,o) 450 MeV $^{129}Xe^{23+}$, and (h,p) 120 MeV $^{197}Au^{9+}$ for 10 μm film thickness of (e–h) C_{60} and (m–p) PTCDA-Cl_4. LET over 12000 eV nm^{-1} is estimated for all the conditions until 3 μm depth.

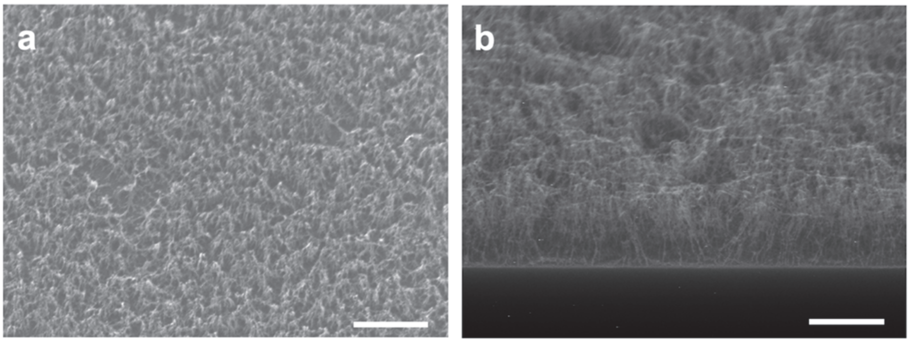

Supplementary fig. 3 | Effect of irradiation fluence on vertical alignment of nanowires. SEM images of nanowires fabricated via charged particle irradiation and subsequent sublimation of C_{60} films with (a) 200 nm and (b) 1000 nm thickness. Scale bars represent 1 µm. Irradiation condition: (a) 350 MeV $^{129}Xe^{26+}$ at 5.0×10^{10} cm^{-2}; (b) 350 MeV $^{129}Xe^{26+}$ at 1.0×10^{10} cm^{-2}.

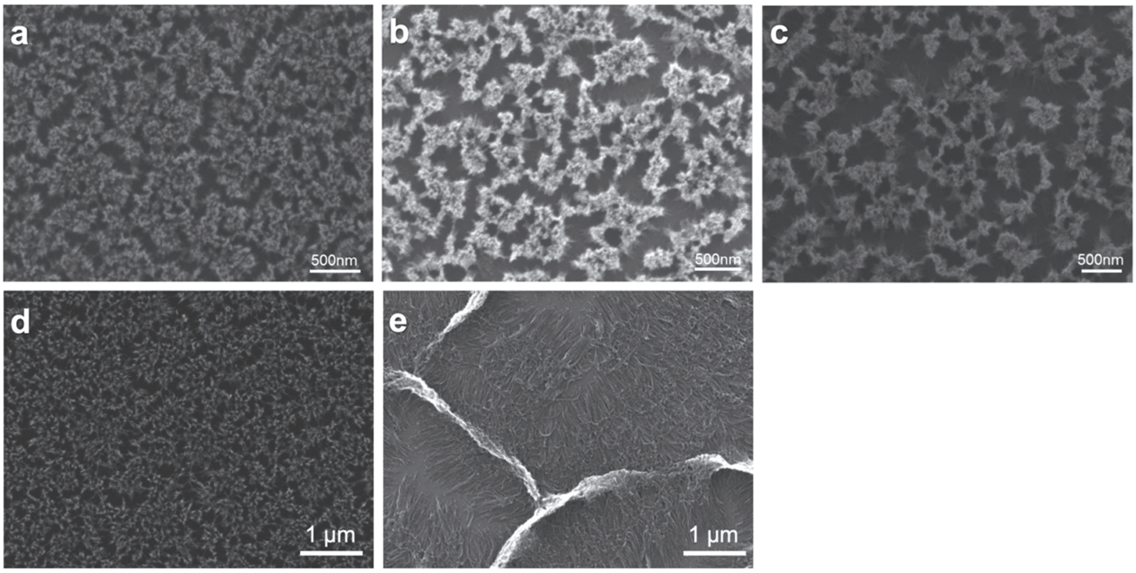

Supplementary fig. 4 | Solvent treatment of isolated standing nanowires. SEM images of standing nanowires fabricated via irradiation of (a) 250 nm and (d) 500 nm C_{60} film thickness and subsequent sublimation. Irradiation condition: (a) 490 MeV $^{192}Os^{30+}$ at 1.0×10^{11} cm^{-2}; (d) 350 MeV $^{129}Xe^{26+}$ at 1.0×10^{11} cm^{-2}. SEM images of C_{60}-based standing nanowires after immersed in 1,2-dichlorobenzene for (b) 5 sec and (c) 5 min. SEM images of C_{60}-based standing nanowires in (d) after immersed in 1,2-dichlorobenzene for (e) 5 min.

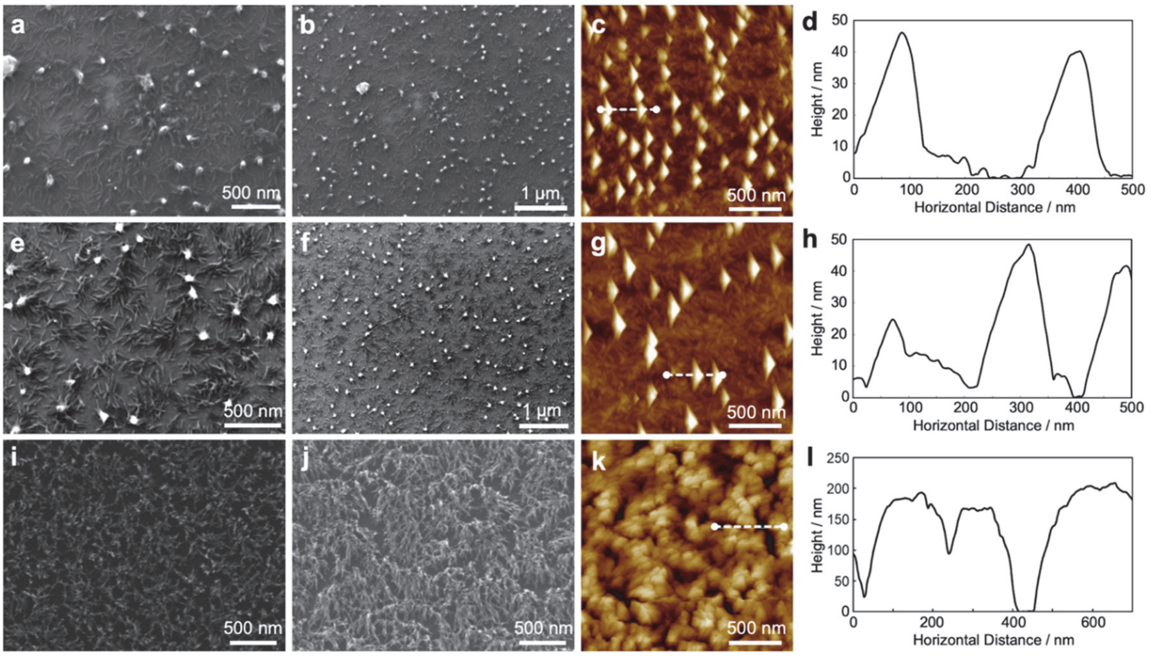

Supplementary fig. 5 | Nanowires from other fullerene derivatives isolated by sublimation. (a,b) SEM images, (c) AFM images, and (d) height profile along white dashed line in (c) of nanowires fabricated via irradiation of $PC_{61}BM$ ([6,6]-phenyl-C_{61}-butyric acid methyl ester) spin-coated film (100 nm thickness) with 350 MeV $^{129}Xe^{26+}$ at 1.0×10^{11} cm^{-2} and isolated by sublimation at ~300 ºC. (e,f) SEM images, (g) AFM images, and (h) height profile along white dashed line in (c) of nanowires fabricated via irradiation of 100 nm thickness $PC_{61}BM$ spin-coated film with 350 MeV $^{129}Xe^{26+}$ at 1.0×10^{11} cm^{-2} and isolated by development with 1,2-dichlorobenzene at 25 ºC. (i,j) SEM images, (k) AFM images, and (l) height profile along white dashed line in (c) of nanowires fabricated via irradiation of 230 nm thickness vapor-deposited C_{70} film with 350 MeV $^{129}Xe^{26+}$ at 1.0×10^{11} cm^{-2} and isolated by sublimation at ~300 ºC.

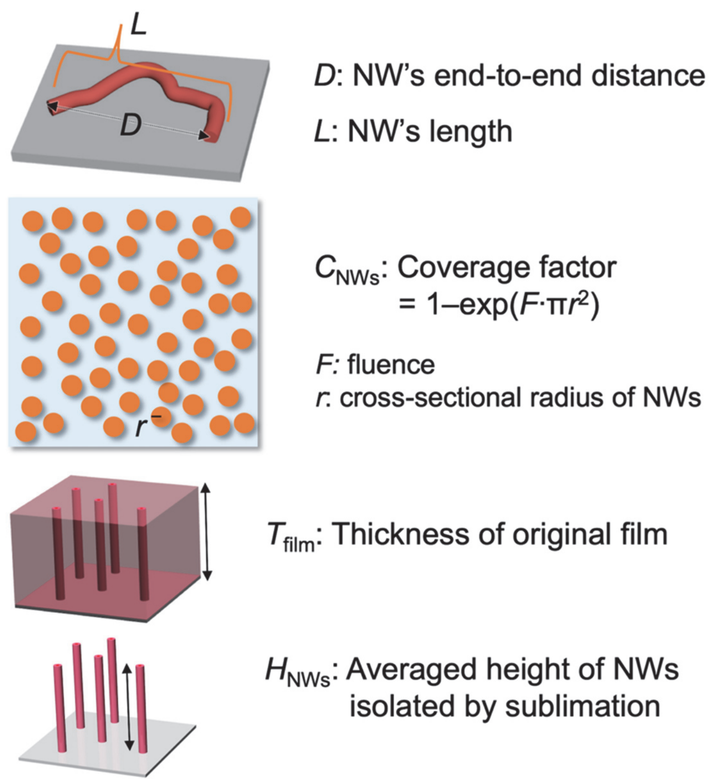

Supplementary fig. 6 | Schematic illustrations explaining parameters used in **Table 1**.

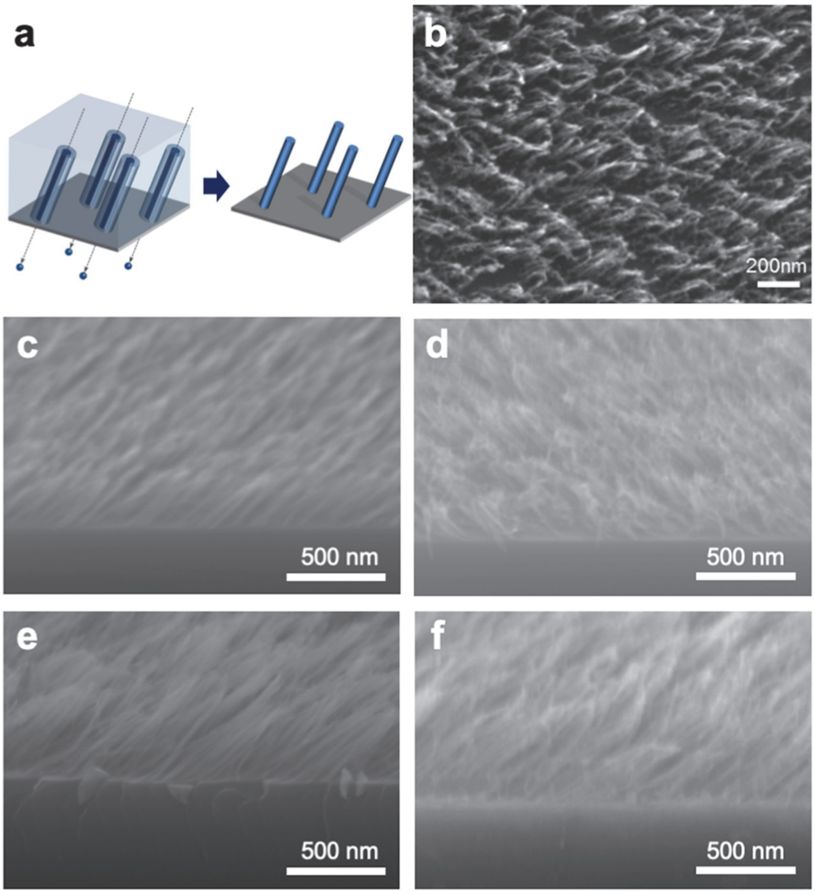

Supplementary fig. 7 | Fabrication of tilted nanowires. (a) Schematic illustration and (b) top views in SEM for nanowires fabricated via irradiation of 200 nm thickness C_{60} film with 350 MeV $^{129}Xe^{26+}$ at 1.0×10^{11} cm^{-2} in oblique direction and subsequent sublimation. Tilt view in SEM for nanowires fabricated via irradiation of (c) 200 nm thickness PTCDA-Cl_4, (d) 200 nm thickness TBP, (e) 450 nm thickness BPEA, and (f) 500 nm thickness DBBA films with 490 MeV $^{192}Os^{30+}$ at 2.0×10^{11} cm^{-2} in oblique direction and subsequent sublimation.

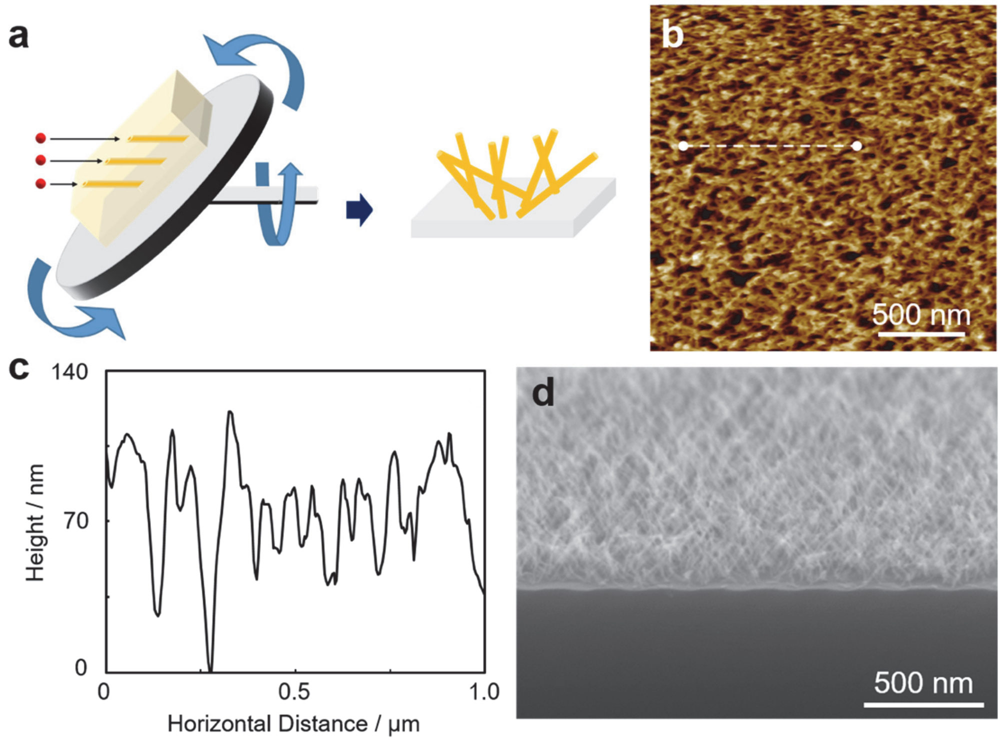

Supplementary fig. 8 | Fabrication of cross-connected nanowires. (a) Schematic illustration of high-energy ion irradiation of thin films on automatically rotating disk. The incident angle between the ion vector and plane of disk was set at 45º. (b) AFM image and (c) height profile along white dashed line in (b), and (d) SEM image of crosslinked nanowires fabricated via irradiation of 200 nm thickness vapor-deposited PTCDA-Cl_4 film irradiated with 450 MeV $^{129}Xe^{23+}$ at 4.0×10^{11} cm^{-2} and isolated by sublimation.

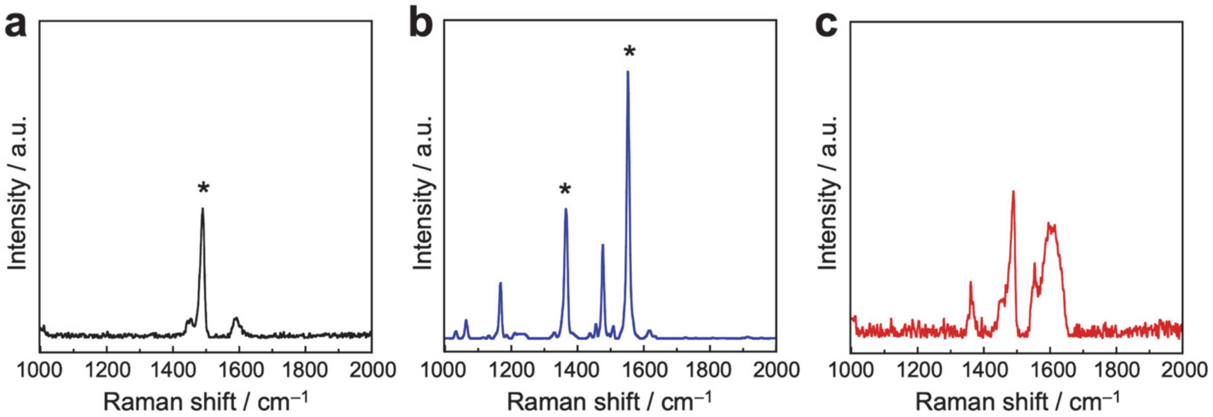

Supplementary fig. 9 | Raman spectra of (a) C_{60} film, (b) CuPc film, and (c) CuPc–C_{60} nanowires on Si substrates. The film of CuPc or C_{60} was prepared by depositing them on Si substrates at 100 nm thick respectively. CuPc–C_{60} nanowires were fabricated by irradiation with 450 MeV $^{129}Xe^{23+}$ ions at the fluence of 5.0×10^{11} cm^{-2}, followed by dry process development. The characteristic peaks represent (a) A_g mode (1470 cm^{-1}) of C_{60}, and (b) A_{1g} mode (1336 cm^{-1}, 1552 cm^{-1}) of CuPc.

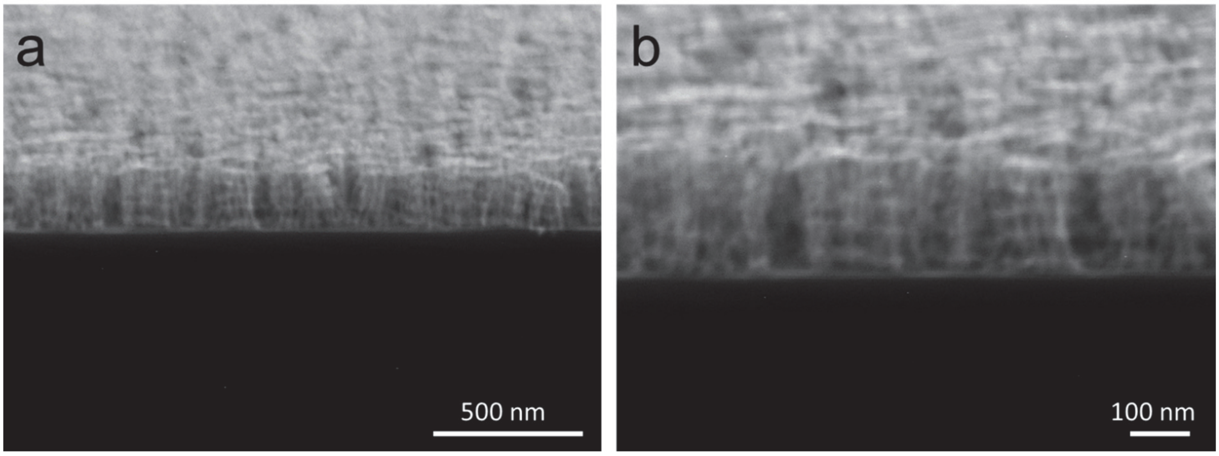

Supplementary figure 10 | Fabrication of multi-segment nanowires. (a,b) SEM images of multi-segment nanowires fabricated by irradiation of 450 MeV $^{129}Xe^{23+}$ to CuPc and C_{60} film with 10 layers (20 nm thick for each layer, total 200 nm thick). Clear bright tone is suggestive of Cu atoms in CuPc layers in contrast to C_{60} layers with only light carbon atoms.

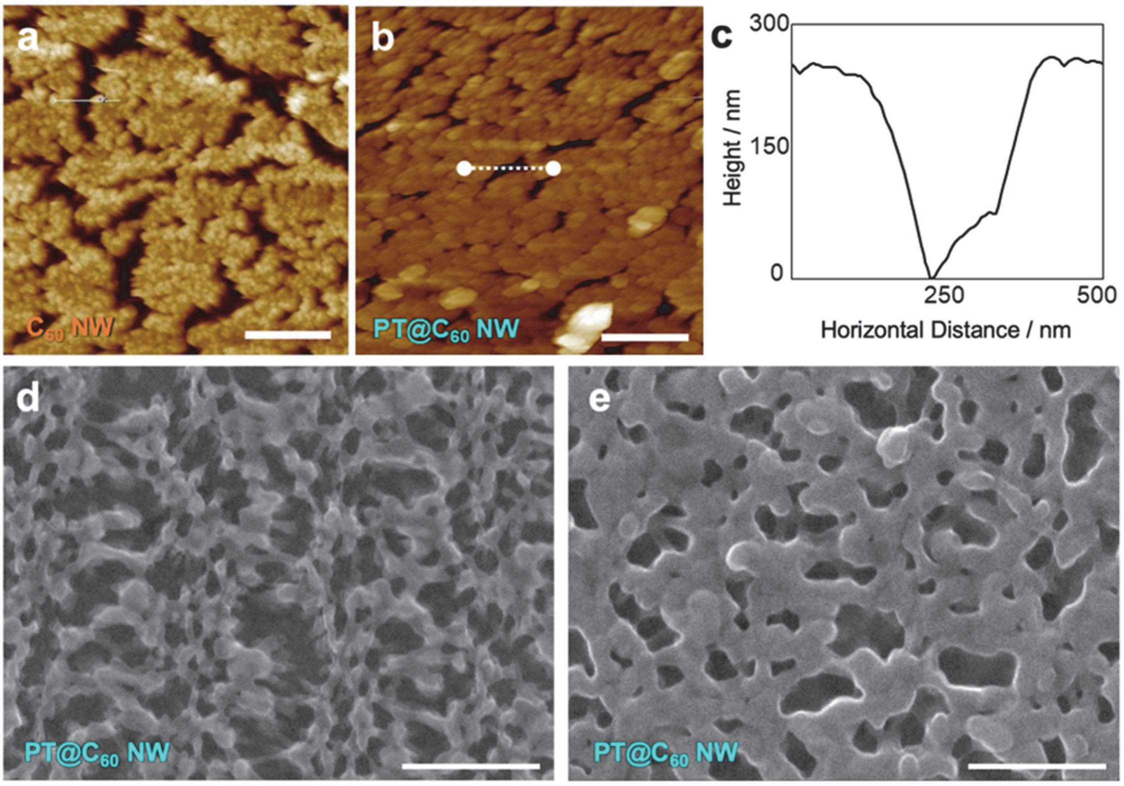

Supplementary fig. 11 | Morphology of coaxial nanowires. AFM images of (a) C_{60}-based standing nanowires and (b) polythiophene-rapped C_{60}-based coaxial nanowires (PT@C_{60}). Height profile along white dashed line in (b). Top views of SEM images for PT@C_{60} after (d) 1 min and (e) 3 min electropolymerization of 2,2'-bithiophene. Scale bars represent 500 nm. Irradiation condition: 450 MeV $^{129}Xe^{23+}$ at 1.0×10^{11} cm^{-2} for C_{60} nanowires.

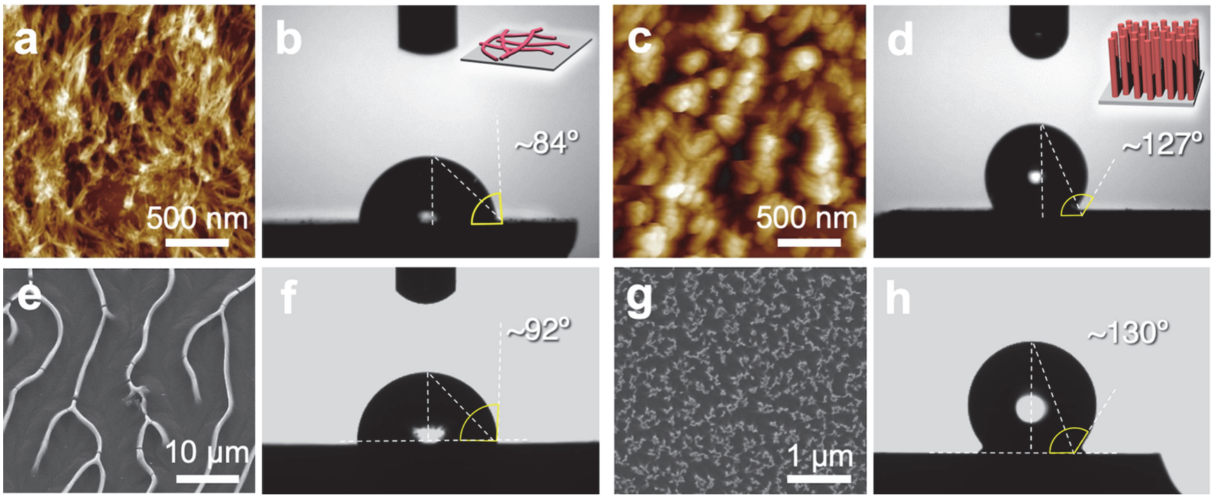

Supplementary fig. 12 | Difference of surface water repellency controlled by orientation of nanowires. AFM top-view images of BPEA nanowires (a) knocked-down on a substrate after development with *n*-decane and (c) standing on a substrate isolated by sublimation. SEM top-view images of C_{60} nanowires (e) knocked-down on a substrate after development with 1,2-dichlorobenzene and (g) standing on a substrate isolated by sublimation. (b,d,f,h) Sectional images of water droplets on nanowire surfaces corresponding to images (a,c,e,g), respectively. The nanowires were fabricated via irradiation with 120 MeV $^{197}Au^{9+}$ at 10^{11} cm^{-2}.

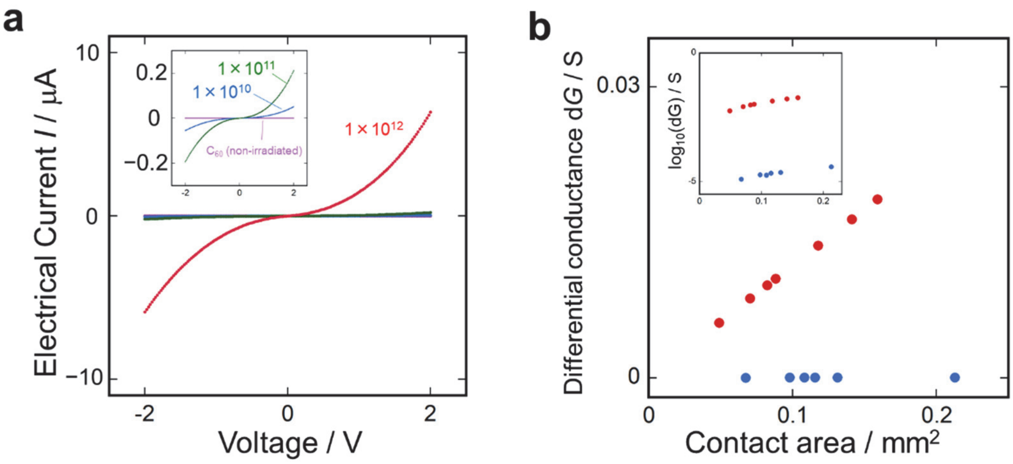

Supplementary fig. 13 | Electrical conductivity of nanowires. (a) *I*–*V* traces of C_{60} nanowire plexus recorded for a variety of number density of nanowires from 1×10^{10} to 1×10^{12} cm^{-2}. The enlarged traces for a non-irradiated control thin film and nanowires with the lower number density were displayed in the superimposed one. (b) Dependence of differential conductance under 1V bias applied on the area of top electrodes for the nanowire plexus with the number density of (blue) 1×10^{10} and (red) 1×10^{12} cm^{-2}. Superimposed figure is a logarithmic plot of the conductance against area of the electrodes.

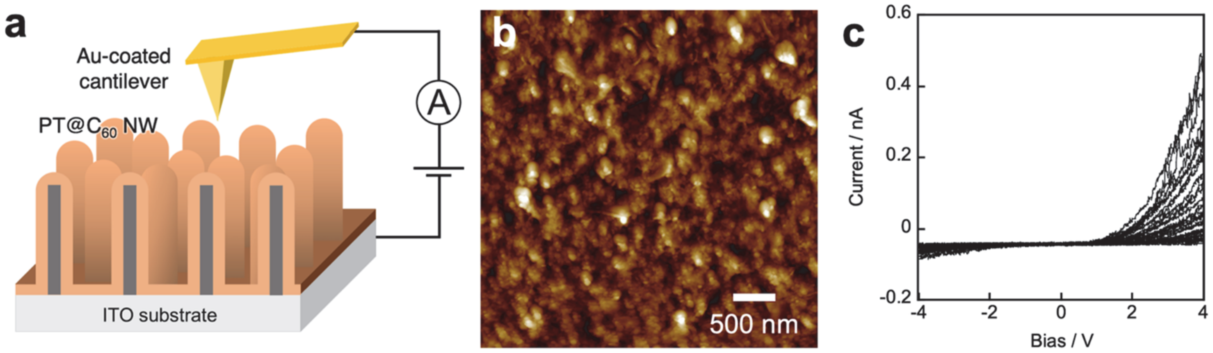

Supplementary fig. 14 | *p-n* heterojunction nanowires as a rectifier diode. (a) Schematic illustration of conductivity measurement of a PT@C_{60} nanowire by contact-mode AFM with an Au coated cantilever. Spring constant of the cantilever was 0.23 N m^{-1}, and the deflection of the cantilever was set at 2.0 nm, acting interfacial force of ~ 100 pN between the cantilever and PT surfaces. (b) Top view of PT@C_{60} nanowires. (c) *I*-*V* traces of PT@C_{60} nanowires measured at 30 distinctive positions in (b).
